# A Comparative View on Human Somatic Cell Sources for iPSC Generation

**DOI:** 10.1155/2014/768391

**Published:** 2014-11-06

**Authors:** Stefanie Raab, Moritz Klingenstein, Stefan Liebau, Leonhard Linta

**Affiliations:** Institute of Neuroanatomy, Eberhard Karls University Tübingen, Österbergstraße 3, 72074 Tübingen, Germany

## Abstract

The breakthrough of reprogramming human somatic cells was achieved in 2006 by the work of Yamanaka and Takahashi. From this point, fibroblasts are the most commonly used primary somatic cell type for the generation of induced pluripotent stem cells (iPSCs). Various characteristics of fibroblasts supported their utilization for the groundbreaking experiments of iPSC generation. One major advantage is the high availability of fibroblasts which can be easily isolated from skin biopsies. Furthermore, their cultivation, propagation, and cryoconservation properties are uncomplicated with respect to nutritional requirements and viability in culture. However, the required skin biopsy remains an invasive approach, representing a major drawback for using fibroblasts as the starting material. More and more studies appeared over the last years, describing the reprogramming of other human somatic cell types. Cells isolated from blood samples or urine, as well as more unexpected cell types, like pancreatic islet beta cells, synovial cells, or mesenchymal stromal cells from wisdom teeth, show promising characteristics for a reprogramming strategy. Here, we want to highlight the advantages of keratinocytes from human plucked hair as a widely usable, noninvasive harvesting method for primary material in comparison with other commonly used cell types.

## 1. Introduction

Since the initial description of Yamanaka and Takahashi in 2006, the generation of induced pluripotent stem cells (iPSCs) has become a widely used method [1]. As human iPSCs are generated without the destruction of an embryo, the disadvantage of broad ethical concerns is diminished. However, the most important advantage of iPSCs compared to ESCs (embryonic stem cells) is the possibility to use mature somatic cells from patients who suffer from genetically defined diseases [2–4]. The obtained iPSCs exhibit the donor's specific genetic changes, opening the possibility to characterize specific phenotypes in patient derived stem cells and their differentiated progeny. The so-obtained differentiated disease specific cells could also be used for drug screenings to find substances which specifically diminish or revert observed phenotypes. These characteristics could pose a powerful tool to better understand a disease pathomechanism [5] and might serve for future therapeutic approaches (reviewed in [6]). The future benefit for patients is that, in transplantation, autologous stem cells, differentiated cells, and even stem cell derived tissues show no relevant graft-versus-host disease.

There are different challenges to cope with achieving an easy, efficient, and fast reprogramming protocol. On one hand, the appropriate reprogramming method needs to be chosen. The most commonly used method is the integration of the reprogramming factors into the genome by lentiviral or retroviral transduction [7, 8]. It is the easiest and most efficient method by now, though in the future other solutions will be more in focus since cells generated by permanent and random integration of exogenous genes have a certain oncogenic potential and are therefore not suitable for use in therapeutic approaches. To avoid the use of integrating viruses, other reprogramming approaches, for example, the use of Sendai viruses [9], plasmids [10], modified RNA [11], or small molecules [12, 13], have been described. Another important issue is the choice of the starting material. Takahashi et al. used fibroblasts as the starting somatic cell type since fibroblasts were also used for reprogramming mouse cells earlier. In addition, fibroblasts are easily cultured and expanded. Nevertheless, some disadvantages of reprogramming fibroblasts such as their relatively low reprogramming efficiency and especially the need of uncomfortable biopsies have led to a search for other cell sources. The molecular properties of the different cell types leading to variations in the reprogramming efficiency have been reviewed in [14]. Publications describe, amongst others, three relatively easy to obtain cell types, that is, blood cells [15], exfoliated renal tubular epithelial cells, obtained from urine [16], and keratinocytes from plucked hair [17]. In particular, keratinocytes seem to be a promising material for reprogramming because they combine the benefits of a noninvasive procedure, an easy way of transport, and a high reprogramming efficiency.

In the current review, we aim to describe in detail the use of hair follicle derived keratinocytes for reprogramming into patient derived iPSCs and discuss the advantages of keratinocytes compared to other starting materials.

## 2. Reprogramming

First, we want to shortly introduce an exemplary reprogramming method, conducted with hair derived keratinocytes as the starting material and lentiviral transduction of the four transcription factors:* OCT4*,* KLF4*,* SOX2*, and* C-MYC*.

**Figure 1:**
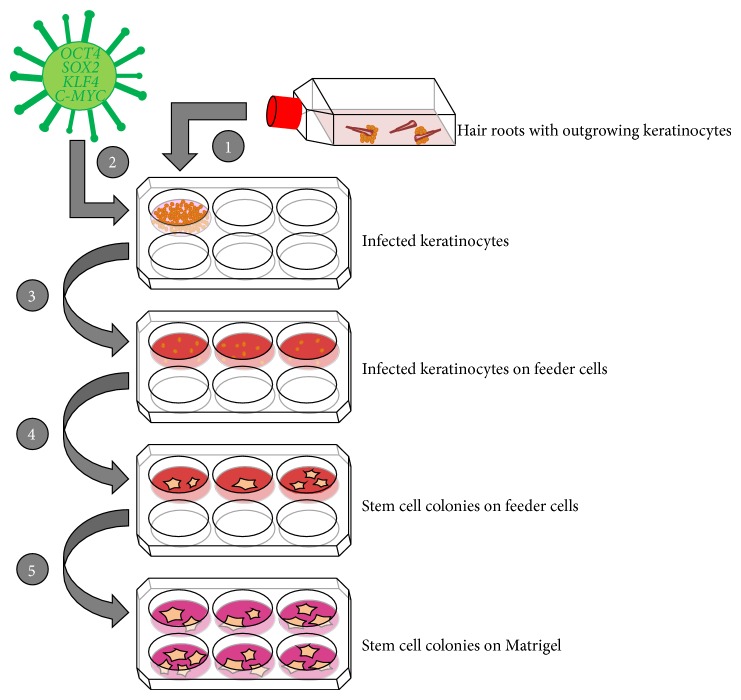
Reprogramming of keratinocytes. (1) Plucked hair is cultured in flasks until outgrowth of keratinocytes. Those are transferred to a six-well plate. (2) Keratinocytes are infected with a lentivirus containing the four reprogramming factors* OCT4*,* SOX2*,* KLF4*, and* C-MYC*. (3) Infected keratinocytes are transferred to a plate with feeder cells (e.g., rat embryonic fibroblasts) in reprogramming medium. (4) After two to three weeks, stem cell colonies arise, and the uninfected keratinocytes do not proliferate in the reprogramming medium. (5) When the stem cell colonies reach a certain size, they are picked mechanically and a feeder-free system may be used for cultivating the human iPSCs.

Keratinocytes are propagated and infected while still in the growth phase. After infection, keratinocytes are transferred onto a feeder layer (e.g., murine or human mitotically inactivated fibroblasts) until primary colonies with an obvious stem cell morphology reach the appropriate size to be picked mechanically and to be subsequently cultured in, for example, a feeder-free system (Figure 1). After testing newly generated cell lines for stem cell characteristics, such as pluripotency marker expression, genetic coherence, and differentiation capacity, iPSCs can be propagated and used for further applications.

The common aim of all reprogramming methods is the forced expression of reprogramming factors, in our example* OCT4*,* KLF4*,* SOX2*, and optionally* C-MYC*. Although C-MYC is not essential for the reprogramming process, it can highly increase the reprogramming efficiency [18]. On the other hand, its oncogenic potential suggests its omission for clinical applications [19].

Various advantages became apparent in recent reprogramming methods compared to genome integrating viruses like retrovirus and lentivirus. The application of non- or low genome integrating and virus free methods harbors a positive safety aspect in the utilization of iPSCs in clinical implications. The translation of iPSC technology into cell therapeutic applications is becoming more and more important [20]. A still existing huge disadvantage of most of these methods is the low efficiency. One promising integration free method is the use of Sendai RNA viruses, although that this system comes along with possible reactivation of viral genes and shows a rather low reprogramming efficiency [9]. Other methods with low genomic integration are plasmid or episomal transfection, but the risk of a host genome integration cannot be completely eliminated. Moreover, also these systems exhibit a low reprogramming efficiency compared to other systems [10, 21]. Another very different integration free method is the direct delivery of reprogramming proteins into the cells [22]. Nevertheless, this approach is technically difficult and requires high amounts of special proteins. Again protein transfection also shows a low efficiency. A very promising tool is represented by transfected mRNA. It is a nonintegrating, virus free method which shows a comparably high efficiency [11]. Due to the fact that repeated transfections are necessary, this method is much more extensive and expensive compared to the viral methods and therefore not yet the common state of art for most applications.

Overall, numerous reprogramming methods exist which all have their own positive and negative aspects (all reprogramming strategies are reviewed in [23]). In particular, the nonintegrating, virus free protocols will become more and more important with the look towards clinical trials. These methods should be not only intensively propagated and further improved, but also be tested with different starting materials. In this review, the reprogramming protocols of the different somatic cells sources are compared on virus-based methods due to their higher efficiency and the well-established processes.

## 3. Epigenetics in Reprogramming

The general reprogramming process changes the transcriptome and chromatin state of the somatic cell to that of a pluripotent cell [24].

There are several issues which are under intensive discussion, like the influence of DNA and H3K9 methylation on the binding of the reprogramming factors in the early phase of the reprogramming process. The hypermethylation status of the* Nanog* and* Oct4* promoters in the starting phase which gain active chromatin modifications in the late phase of the reprogramming is very well analyzed examples [25, 26]. There are other very important points which have to be considered with respect to reprogramming such as possible transition between reprogramming steps or the status of the X chromosome and its inactivation while reprogramming, all of which are reviewed in [27].

With a special eye on the somatic primary cell source, several studies are published depicting differences with respect to their epigenetic features. The so-called “epigenetic memory” describes the inheritance of the initial epigenomes and transcriptomes of the primary somatic cell type to the iPSCs. This means that aberrations acquired during reprogramming, like impaired functioning of imprinted genes, genetic instabilities, aberrant patterns of DNA methylation, or changed numbers of gene copies [28–30], as well as markers (like unique DNA methylation signatures) of the origin somatic cell, are inherited to the iPSCs [31, 32].

The main consensus of the publications dealing with epigenetics and iPSCs is that differentiation of iPSCs is primed to their original cell state meaning fibroblasts-derived iPSCs differentiate preferably into osteogenic direction whereas iPSCs from blood cells form more hematopoietic colonies (reviewed in [33]). However, several groups describe a loss of the epigenetic memory after prolonged iPS cell culture which leads to the conclusion that the primary cell source may not be absolutely essential for redifferentiation [34, 35].

## 4. What to Do with the Generated iPSCs?

A variety of publications support the hypothesis that iPSCs generated from different primary cell sources share most characteristics with embryonic stem cells [7, 16, 17, 36, 37]. Immunofluorescence staining, methylation assays, teratoma formation assays, karyotyping, or bisulfite genomic sequencing are only a selection of methods commonly used to prove the potential of differentiation into all three germ layers and pluripotency capacity of the generated iPSCs. Hence, no significant differences were published concerning the primary cell source and their respective behavior as reprogrammed stem cells, although a variety of divergences are observed between iPS clones.

One of the major applications of iPSCs is the differentiation into specific cell types. Numerous protocols have been established to generate tissue specific progenitor or mature cells involving all three germ layers. This includes, amongst various other cell types, for example, cardiac muscle cells, endodermal progenitor cells [38], and photoreceptor cells of the retina [39] or specific neuronal subtypes. IPSCs are used to study differentiation into exotic cell types and their underlying differentiation mechanisms but they are also utilized for* in vitro* models to investigate a variety of diseases. The most desired goal and hope is the generation of patient specific iPSCs and further retransplantation of autologous cells into the malfunctioning organ. One of the benefits of using autologous cells is the circumvention of a graft-versus-host reaction. Furthermore, from an ethical point of view, iPSCs avoid the use of the controversially discussed embryonic stem cells, at least in many countries.

In August 2013, the first human pilot safety clinical trial using these autologous iPSCs has been launched in Japan. Here, fibroblasts were used as a somatic cell source from patients suffering from wet age-related macular degeneration. This disease is marked by blood vessels growth up from the choroid behind the retina, which can lead to a detachment of the retina and causes the loss of vision in the centre of the visual field (macula). The mostly elderly patients suffer from visual impairment, although enough peripheral vision remains [40].

In this setting, the patient derived iPSCs were differentiated into a monolayer of retinal pigment epithelial cells and further transplanted into the affected retina. Several preceding* in vitro* studies as well as the approval of the method in animal models had been performed [41–43]. The targeted time-frame of this study will last approximately 10 months with a follow-up study of four years. More clinical trial will and should follow in this promising field of research.

## 5. Fibroblasts as the Common First Choice

To produce human iPSCs from somatic tissue, different starting cell types are available. In general, each actively dividing somatic cell type can be used for reprogramming [44]. To date fibroblasts are still the most commonly used primary cell source. Nevertheless, the question that remains is as follows: why they are still considered as the first choice when it comes to reprogramming mature cells to iPSCs?

Besides their cheap and easy handling, a lot is known about fibroblasts and they are well established in several fields of research. The first successful approach of reprogramming adult cells has been performed using mouse fibroblasts [1]. When this method was adapted to human cells, the first attempt was consequentially done with fibroblasts. As other groups started to reproduce this groundbreaking achievement, most protocols were also using fibroblasts before new techniques were investigated. Nevertheless, every new method can be improved, and since the starting material is a crucial variable in the whole reprogramming process, many groups have started to search for better alternatives as there are some disadvantages coming up with the use of fibroblasts. Fibroblasts are mesenchymal cells within the dermis layer of the skin and are responsible for producing precursor molecules, parts of the extracellular matrix. With respect to the generation of patient specific iPSCs, in most clinical approaches, it is nearly indispensable to harvest cells directly from the affected patient. Most commonly, normal human dermal fibroblasts are obtained either from adult skin biopsies or neonatal foreskin biopsies from circumcisions. In case of adult patients, dermal fibroblasts can be established from a skin punch biopsy with the common punch size of 3.5 or 4 mm. This method is very painful; hence, the selected position, mostly located at the arm, has to be anaesthetized. Secondary effects of skin punch biopsies are small bleedings and punch biopsies with a big diameter have to be stitched [45]. Furthermore, a certain risk of infection, allergic reactions to the anesthetic, and the formation of scar tissue or keloids are present. Taken together, a skin biopsy is not a simple intervention and has to be supervised and performed by medical professionals.

Alternatively, fibroblasts are commercially available from several companies. Meanwhile, human fibroblasts from various organs and tissues of the body are available, for example, dermal [46], cardiac (ventricle or atrium) [47], lung [48], or periodontal ligament fibroblasts [49]. Even cells from patients who suffer from different diseases are available such as fibroblasts isolated from lung tissue of asthma patients, chronic obstructive pulmonary disease, or cystic fibrosis [50]. However, fibroblasts from donors with rare and exotic diseases are hardly available.

Fibroblasts start their outgrowth from the tissue piece within one to two weeks and are very easy to cultivate with a minimum of serum and medium [51]. The low methylation status of the promoter regions of* OCT4* and* NANOG* in fibroblasts as well as the resulting low endogenous expression of these factors may be involved in the good reprogramming ability associated with transcriptional and epigenetic states favorable to reprogramming [51]. Depending on the fibroblast subtype, they exhibit a very high proliferation rate compared to other cell types. This looks like an advantage at first but also has disadvantages. Not reprogrammed fibroblasts bear the risk to overgrow the newly reprogrammed cells, hinder their proliferation, and deplete growth factors in the medium. However, for an efficient reprogramming process, skin fibroblasts should only be used at a very low passage, not higher than passage five [51]. With higher passages, the reprogramming efficiency is reduced and the risk of accumulated genomic alterations is increased.

The main limiting hurdles of reprogramming are the timeframe and the efficiency. The percentage of positively reprogrammed fibroblasts is stated at about 0.01–0.5%, which is quite low. It also takes a longer time period until stem cell colonies appear after infection compared to other cell types [7, 17]. The whole reprogramming process needs approximately three to five weeks [7]. In contrast, keratinocyte derived iPSC colonies reach the appropriate size for passaging already after 2-3 weeks. There are several hypotheses for the low efficiency rate and the rather long time period for reprogramming fibroblasts.

One possible reason could be the mesenchymal-to-epithelial (MET) transition. MET is a crucial step in normal embryogenesis and early development. Accordingly, it also occurs in the reprogramming process, mimicking these developmental steps* in vitro*. During reprogramming, three steps can be distinguished, namely, initiation, maturation, and stabilization [52]. MET is mainly present in the initiation phase of fibroblast reprogramming which precedes the upregulation of pluripotency markers. In the beginning, first epithelial-associated genes like* EPCAM* (epithelial cell adhesion molecule) or epithelial junctional protein E-cadherin (*CDH1*) are induced. In addition, transcription factors which maintain the mesenchymal phenotype by directly repressing epithelial gene expression are themselves repressed. This facilitates the switch from the mesenchymal to the ectodermal germ layer gene expression [52]. Keratinocytes are of ectodermal origin so no MET has to be passed which could have positive effects on the reprogramming efficiency and speed.

## 6. Blood as an Alternative

When generating iPSCs from blood cells, it has to be distinguished between the use of peripheral blood and umbilical cord blood. In case of using adult peripheral blood, there are two options for harvesting the starting material for reprogramming. One way is to use mobilized CD34^+^ peripheral blood cells isolated in a process similar to the procedure which is routinely performed in stem cell donation [15]. In the preparation phase, the donor has to inject himself the granulocyte colony-stimulating factor (G-CSF), a growth factor which is commonly present in the human body, on five consecutively following days [53]. The process before the actual isolation of the starting material is time-consuming, cost intensive, and painful for the cell donor. In the human body, G-CSF is strongly involved in the regulation of the hematopoietic system, particularly stimulating the production of white blood cells. The overdose of this cytokine leads to an increase in the production and release (or mobilization) of hematopoietic stem cells from the bone marrow into the peripheral blood. In a process called aphaeresis, the CD34 positive stem cells can be separated from blood in a four-hour process while the cell donor is connected to a machine via flexible tubes with its blood flowing through [54]. The following day, the procedure often has to be repeated to isolate the residual stem cells. There are some adverse effects related to the use of G-CSF like bone pain, headache, fatigue, or nausea which makes the whole procedure even more uncomfortable for the patient [55]. The highly time-consuming and expensive aphaeresis shows another disadvantage of using blood cells as an appropriate somatic cell source. Additionally, the mobilization of the peripheral blood cells can only be done with healthy people because of the external administration of the cytokine. This is another severe drawback. Nevertheless, the isolated hematopoietic stem cells from peripheral blood can be further cultivated and reprogrammed to iPSCs which show the same characteristics as other iPSC lines or embryonic stem cells [15].

Another less invasive method to obtain cells from peripheral blood is to isolate specific mononuclear cells. The isolation of these cells can be conducted via density gradient centrifugation. Finally, mature T-cells and myeloid cells are purified and can be used for further reprogramming. But, from the current stand of research, it is not known if iPSCs generated from peripheral blood T cells differentiate normally. They have preexisting V(D)J rearrangements at the T-cell receptor loci which could lead to the development of T-cell lymphomas [56]. Also, the reprogramming efficiency of these cells is ten to fifty times lower compared to human fibroblasts [57].

Almost all studies using blood as primary cell source required blood samples in an average range of 10 mL. Two publications described the use of smaller amounts of peripheral blood (2–6 mL) for the purification of enough CD34^+^ cells for further successful reprogramming [58, 59]. An even better solution is the generation of iPSCs from human finger-prick blood. In a recently published paper, the authors could prove that the volume of a single blood drop is sufficient for the isolation of enough cells for successful reprogramming [60]. They could even show that this single-drop volume (10 *μ*L) is sufficient for reprogramming, DNA sequencing, and blood serotyping in parallel. This easy and barely invasive method to get somatic cells for reprogramming is not only uncomplicated for the patients, as they can even sting their fingers themselves, but also comes along with a high reprogramming efficiency (100–600 colonies per mL of finger-tip capillary blood). The development of international iPSC banks can be facilitated by this protocol.

Beside the use of hematopoietic stem cells or mature T-cells from peripheral blood, human umbilical cord blood represents a further somatic cell source. Endothelial cells from cord blood build up a thin layer of cells, the epithelium, which lines the interior surface of blood, and lymphatic vessels [61]. The isolation of epithelial progenitors from cord blood is ten times more efficient than from adult peripheral blood, which indicates that cord blood is more favorable as primary cell material. Another great advantage is that cord blood derived cells are a very “young,” and therefore fewer nuclear and mitochondrial mutations are present at the time point of cell harvesting and conservation [44]. Certainly, the procedure of harvesting and conservation of umbilical cord blood is highly expensive and needs to be performed directly after birth.

## 7. Urine: A Noninvasive Method

Isolated cells from urine are mostly exfoliated renal epithelial cells, which are excreted in a normal process of detaching into urine daily. Therefore, 50 to 200 mL urine has to be collected in the middle stream of the micturition. After consecutive washing and centrifugation steps, the isolated epithelial cells can be taken in culture [62]. More precisely, these cells are squamous cells from the urethra with a defined epithelial phenotype. Urine cell derived iPSCs show similar expression and pluripotency patterns compared to embryonic stem cells or iPSCs from other sources [63]. The collection of urine is noninvasive with no need of medical personnel. This brings very few indispositions for the patient. Urine samples are easily accessible because they are often routinely collected for clinical diagnosis and independent of age or sex. The reprogramming efficiency lies in the range of 0.1 to 4%, which is much higher than that of fibroblasts or blood cells [16]. Urine cells can be frozen and thawed for several times without decreasing the reprogramming efficiency. Nevertheless, they show a reduced reprogramming efficiency after five passages [62]. To summarize, the obvious advantage of collecting urine is the easy and noninvasive handling and the age and gender independent availability.

## 8. Keratinocytes: The Chosen Ones

Using keratinocytes comes along with a lot of improvement and few disadvantages. First, we want to introduce the source of keratinocytes. There are different types of human hair, vellus, and terminal hair. Beside these two types of hair, there is the so-called Lanugo hair which usually appears only on the body of a fetus or newborn baby [64]. The change from vellus to terminal hair is androgen dependent. Thus, men exhibit more visible terminal hair than women. Starting with the puberty, due to the hormone modulation, terminal hair begins to replace the vellus hair. The crucial difference between these two types of hair is that terminal hair is more pigmented and thicker and it is connected to sebaceous glands [65]. For the establishing of keratinocyte cultures from hair follicles both, fine vellus as well as thick terminal hair can be used as the starting material.

There are different types of hair on the human body which are recommendable for generating iPSCs. Besides scalp hair, other facial hair types like beard, eye brown, or hair from the nose can be used. The specialty of these hair subtypes is that follicles continuously produce thick and pigmented terminal hair unaffected by androgens. These types of hair exist as terminal hair since childhood and the composition and growth are triggered hormonally.

**Figure 2:**
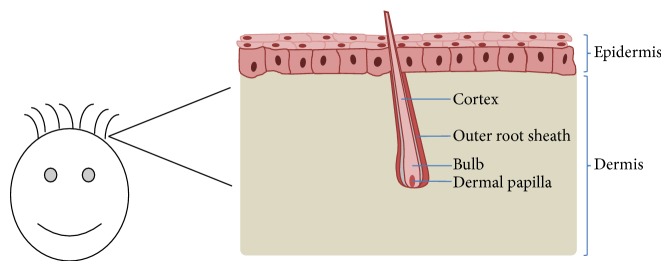
Structure of a hair follicle embedded in skin. Human plucked scalp hair lies in the dermis. Under culture conditions, keratinocytes will grow out of the outer root sheath.

Keratinocytes account for most of the cells in the epidermis of the human skin. The epidermis is situated directly above the dermis, separated by the basement membrane. Dermal keratinocytes build up the stratifying epithelial tissue although other cell types like melanocytes are additionally found in this area. Here, they build four macroscopically discriminable layers from which they differentiate. Proliferation proceeds upwards from the basal layer (*stratum basale*) via* stratum spinosum* and* stratum granulosum* to the* stratum corneum* where these keratinocytes assemble a constant barrier of the skin. In this proliferating process, the dermal keratinocytes terminally differentiate whereby their nuclei and organelles collapse [65]. To ensure the constant proliferation and the integrity of our skin, multipotent stem cells can rebuild the dermal keratinocytes [66]. Beside dermal occurrence, keratinocytes are also present in hair follicles. Hair follicles are embedded in the dermis and are not visible on the surface of the skin.  Figure 2 shows the structure of a normal human hair. The invisible part of the human hair is enclosed by two root sheaths, the inner (IRS) and outer (ORS) root sheaths. Hair follicle stem cells lie in the bulge of the ORS and give rise to keratinocytes and other cell types [67, 68]. The main task of mature keratinocytes is to produce keratin for the growing hair. Human hair cycles through distinct stages, which can be partially discriminated by the shape of the hair bulb. Darkly pigmented hockey stick shaped bulbs with distinct root sheaths typify anagen hair bulbs which build up new hair roots in the growth phase. Nearly all hair (85%–90%) shows this state. Telogen hair has no or an insufficient root sheath, a club-shaped bulb and is less pigmented than anagen bulbs. At this stage, the hair follicle is resting and regenerating for the production of a new hair. The catagen hair bulb indicates a very short transition phase between the two previous ones where the bulb is narrowing and the hair is atrophying [69].

**Figure 3:**
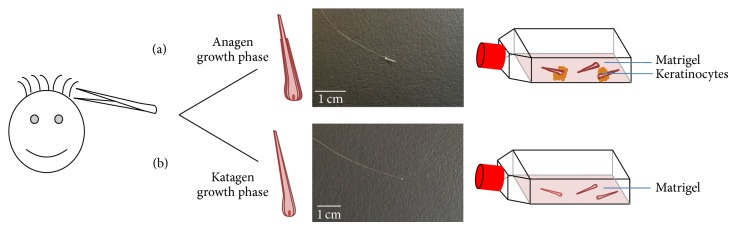
Comparison between plucked hair with good and bad roots. (a) Plucked hair with outer root sheath in the anagen growth phase transferred to a Matrigel coated culture flask. Keratinocytes grow out after 3–7 days. (b) Plucked hair without outer sheath root in katagen growth phase. No keratinocytes will grow out.

To start an efficient reprogramming, an appropriate amount of cells, in this case keratinocytes, is needed. Therefore, it is necessary to obtain a certain expertise in culturing keratinocytes from plucked hair. This has shown to be a drawback for the use of this cell type in various labs. The most important part is plucking the hair with an adequate root, including that the root has to be plucked with an intact ORS (Figure 3). The ORS is visible as a white covering around the root. Once plucked, hair can be stored in normal DMEM medium for several days at room temperature. This implies that hair can be shipped easily from all over the world without fearing the loss of the ability of keratinocytes to proliferate. The convenience for all the involved parties is a huge advantage of this method. The donors do not have to suffer from clinical and surgery investigations, they do not even have to go to hospital. The scientists can obtain the hair at any place with only little effort of the person plucking the hair. Compared with other methods, only urine samples can be obtained as easy as plucking hair. Nevertheless, solely plucked hair can be transported as is before a primary culture is needed.

**Figure 4:**
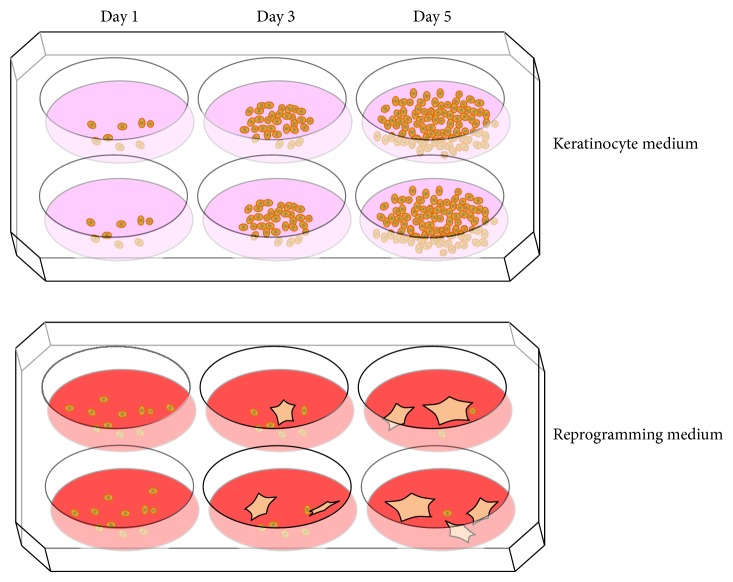
Culture conditions of keratinocytes in different media. (a) Keratinocytes proliferate over time in a low-calcium keratinocyte medium. (b) In reprogramming medium, the keratinocytes stop proliferating and get senescent while iPSCs form colonies and grow.

For culturing keratinocytes, special low-calcium medium formulations are available to prevent the cells from getting senescent too early [70]. This is a benefit because on one hand most other cells do not proliferate well in the low-calcium medium and, on the other hand, after starting the reprogramming, a new medium with normal calcium amounts is used whereby all uninfected keratinocytes reduce or stop proliferation. Only infected cells continue to proliferate, a prerequisite to reprogram into stem cells (Figure 4).

Even with the low-calcium medium keratinocytes isolated from plucked hair differentiate to a senescent state after a few passages or when grown overconfluent. Therefore, it is recommended to start reprogramming with low passages, ideally before passage four.

Once the culture handling has been established, it does not matter if hair from old or young, sick or healthy, male or female donors is used. One outgrowing hair root is enough to obtain enough keratinocytes for a successful reprogramming. Our group has already obtained keratinocytes from newborn babies as well as from older people, both healthy persons and patients who suffer from different genetic diseases. All the volunteers who gave hair samples were pleased that the procedure is noninvasive and does not take longer than a few minutes.

Keratinocytes are reprogrammed faster, approximately in 1-2 weeks in contrast to the duration of 3-4 weeks for fibroblasts. In addition, fibroblasts show much lower expression of* C-MYC* and* KLF4* compared with other cell types [71, 72]. An already high base level of these genes, as observed in keratinocytes, may contribute to a more efficient reprogramming process. Keratinocytes have been described to exhibit a 100-fold higher reprogramming efficiency (about 1-2%) compared to fibroblasts [17, 73]. A successful reprogramming strategy using episomal vectors was recently described, making keratinocytes even more interesting in the future of iPSC generation [74].

## 9. “Exotic” Cell Sources

In addition to the recently described noninvasive preparation of keratinocytes from hair roots and small volume blood from finger pin prick, the use of buccal cell swabs is a simple method to obtain primary starting material. The human oral mucosa consists of a stratified squamous epithelium at the surface and an underlying connecting tissue (lamina propria). The epithelium in the inner cheek is not keratinized and is composed of epithelial cells, pigment cells, Langerhans cells, and Merkel cells [75]. Up to date, no paper has been published directly describing the generation of iPSCs from cheek swabs. But nonetheless the use of buccal swabs is routinely done for the collection of DNA samples, for example, human leukocyte antigen testing [11] or paternity tests. The donor has to brush a swab against the cheek and the loose buccal cells stick to the swab. In this process, cells isolated from the swabs will be taken in culture and expanded and DNA can be isolated.

As any somatic cell can be reprogrammed, it is also theoretically possible to use cells isolated from buccal cell swabs as primary starting material for reprogramming [11].

Several studies were published describing the use of some more exotic cell types. Multipotent mesenchymal stem cells (MSCs) can be relatively easy isolated from mononuclear cells from bone marrow, although a surgical intervention is needed [76]. Approximately one week after bone marrow aspiration, MSCs can be observed and enriched in cell culture. These cells share several properties with fibroblasts, namely, adherent growth and a fibroblast-like morphology [51]. The reprogramming efficiency was indicated at about 0.01%. With respect to the invasive acquisition, the use of MSC is not a broadly applicable choice but represents an option under special circumstances. For example, MSCs can be gathered during planned bone marrow biopsies.

Another possible somatic cell line for reprogramming is primary hepatocytes [37]. This cell type is of endodermal origin and may also be gathered during diagnostic biopsies or surgical interventions. The generation of iPSCs from primary hepatocytes and the following differentiation into cells which are involved in liver specific diseases may be beneficial due to the fact that no germ layer transition between the origin somatic cell and the differentiated liver specific cells generated from iPSCs needs to occur. However, the favoring of differentiation into cell types related to the source cell type for reprogramming is still debated [51]. Subsequently, for example, hepatic progenitor cells or even mature hepatocytes can be further analyzed for drug testing, liver disease modeling, or even therapeutic approaches [37, 77]. The use of patient hepatocyte-derived iPSCs becomes important under distinct circumstances, when somatic mutations are present only in liver cells (this would account also for other organs). This would open the possibility to analyze the disease controlled with mutation-free skin fibroblast from the same patient [37]. It has been published that the reprogramming speed of hepatocytes is faster compared to fibroblasts, blood cells, or bone marrow cells [37]. Another group describes the reprogramming of analogous human pancreatic islet beta cells to iPSCs that can be easily differentiated into insulin-producing cells. This differentiation method, independent from the cell source, may give new possibilities in the treatment of diabetes. However, the reprogramming of this starting material shows an extremely low efficiency (0.0001%) [31].

The generation of human iPSCs from osteoarthritis patient derived synovial cells has been published in 2011 [78] and the use of human third molar mesenchymal stromal cells (wisdom teeth) one year earlier [79].

The question that remains is as follows: for what practical reason new cell sources should be explored? One possible reason is certainly the scientific curiosity to see if the reprogramming of a given cell type is possible and to find cellular characteristics that increase or decrease reprogramming efficiency, a prerequisite to understand pluripotency. Furthermore, it is of broad interest to find new possible cell sources for reprogramming with respect to availability and efficiency. Additionally, it is also advisable to adapt the method to the cell donors. For example, people with burned skin are not recommended for skin biopsies and patients who suffer from leukemia or immunodeficiency should not donate blood cells or have any kind of surgery. On the other hand, for diseases with a genetic background and a phenotype in certain cell types or organs, it can be reasonable to take especially those cells in culture which are affected.

## 10. Summary: Advantages and Disadvantages of Hair Keratinocytes

Human iPSCs can be generated from cells of all three germ layers. The most commonly used cell types are mesodermal fibroblasts or blood cells, hepatocytes of endodermal origin, or ectodermal keratinocytes. All somatic cells described here were used as the starting material for reprogramming and derived iPSCs show high similarity with embryonic stem cells in terms of morphology, gene expression, and renewal and differentiation capacity.  Table 1 summarizes the pros and cons of the major starting materials for reprogramming.

Keratinocytes are convincing in handling, efficiency, and most important in convenience for both patients and scientists. Therefore, we suggest that keratinocytes are a highly recommended starting cell type for iPSC generation in a wide range of research fields.

## 11. Conclusion: Why Does Not Everybody Change to Keratinocytes?

Here, we want to sum up the hard facts on the different primary somatic cell sources. Realistically, most laboratories will most probably stay with their method of reprogramming, using the established somatic cell type. Skin fibroblasts will certainly maintain their position as the most commonly used cells. This is due to the fact that these cells are (1) cheap and easy in their maintenance, (2) have a high viability and culture stability over 5–10 passages, and (3) although they have a low reprogramming efficiency, they will mostly facilitate a successful iPSC generation. In addition, (4) the adaptation to almost all integration free methods has been broadly investigated, and (5) most iPSC banking has been performed up to now with this cell type.

Blood cells from umbilical cord or peripheral blood are (1) much more difficult as well as cost intensive in the initial purification process, (2) the culture conditions of the peripheral blood cells are not stable for long passaging periods, and (3) the reprogramming efficiency is quite low, whereas (4) viral and integration free methods were successfully tested in this system and (5) blood banking could be easily combined with iPSC banking.

Keratinocytes are (1) easy to handle, but relatively cost intensive due to special culture media and surface coatings and are (2) stable only up to about 5 passages. (3) Reprogramming efficiency is very high and (4) adaptable to integration free reprogramming and (5) patient material and iPSC banking can be easily performed.

## Figures and Tables

**Table 1:** Comparison of different starting cell types.

Starting cell type	Hair	Blood			Skin	Urine
Source	Hair follicle	Peripheral blood	Cord blood	Finger pin prick	Skin biopsy/foreskin	Urine
Cell type	Keratinocytes	Mature T-cells Myeloid cells	Endothelial cells	Capillary blood cells	Fibroblast	Renal tubular cells/epithelial cells
Germ layer	Ectoderm	Mesoderm	Mesoderm	Mesoderm	Mesoderm	Mesoderm
Cultivation	+	+	+	+	++	+
Isolation	++	+	+	+	−	++
Reprogramming efficiency	++	−	+	+	+	++
Invasiveness	No	Yes	(Yes)	(Yes)	Yes	No
Availability	++	+	−	++	+	++
Clinical education needed	No	Yes	Yes	No	Yes	No
Selectivity under culture conditions	++	+	+	+	−	−
Reprogramming time	2-3 weeks	3–5 weeks	2–4 weeks	2-3 weeks	3–5 weeks	3 weeks
Culture costs	+	−	−	+	++	+
Integration free methods tested	+	+	+	+	+	+
Direct transport of primary material	+	−	−	+	−	?

Note: ++: very high convenience; +: high convenience; −: low convenience.
